# EFFECTS OF EXERCISE WITH MUSIC IN FRAIL OLDER ADULTS: A SYSTEMATIC REVIEW AND META-ANALYSIS

**DOI:** 10.1093/geroni/igae098.2757

**Published:** 2024-12-31

**Authors:** Namhee Kim, Gwang Suk Kim, Min Kyung Park, Layoung Kim, Jae Jun Lee

**Affiliations:** Yonsei University, Wonju, Kangwon-do, Republic of Korea; Yonsei University, Seoul, Republic of Korea; Mo-Im Kim Nursing Research Institute, Yonsei University College of Nursing, Seoul, Republic of Korea; Yonsei University, Seoul, Republic of Korea; Yonsei University, Seoul, Republic of Korea

## Abstract

Accumulating research on the impact of exercise with music on the health of frail older adults emphasized the necessity of a comprehensive analysis that encompasses physical and emotional health outcomes. Consequently, this study aimed to identify the contents and characteristics of exercising with music and to evaluate the effects of exercising with music on the physical and emotional health of frail community-dwelling older adults. This was a systematic literature review and a meta-analysis. All searches were conducted from April 12, 2022, to March 2, 2024, with no restrictions on language or publication date. Seven electronic databases were searched: MEDLINE, CINAHL, Embase, Cochrane Library, PsyINFO, Google Scholar, and the Virginia Henderson International Nursing Library. PRISMA guidelines for systematic reviews were followed. After screening the initial 1409 studies, 17 studies, including 13 randomized controlled trials and four quasi-experimental studies, were analyzed. The findings indicate that exercise with music has positive effects on reducing frailty (SMD = -0.43, 95% CI = -0.81 – -0.05), improving Timed Up and Go test results (MD = -3.76, 95% CI = -6.67 – -0.85), increasing handgrip strength (MD = 2.01, 95% CI = 0.36 – 3.65), and decreasing depression levels (SMD = -0.34, 95% CI = -0.56 – -0.11) among frail older adults. By addressing both physical and emotional health, exercise with music can significantly contribute to enhancing older adults’ quality of life and reducing the burden of frailty. In conclusion, this review emphasizes musical exercises’ potential as a multifaceted intervention for frail older adults.

